# Atypical Calciphylaxis Following Parathyroidectomy and Persistent Severe Hypocalcemia in a Hemodialysis Patient: A Case Report

**DOI:** 10.7759/cureus.98072

**Published:** 2025-11-29

**Authors:** Christopher H Goss, Geethika Earthineni, Vishwajeeth Pasham, Tibor Fülöp

**Affiliations:** 1 Nephrology, Medical University of South Carolina, Charleston, USA; 2 Pathology and Laboratory Medicine, Medical University of South Carolina, Charleston, USA; 3 Medical Services, Ralph H. Johnson Veterans Affairs Medical Center, Charleston, USA

**Keywords:** adynamic bone disease, calciphylaxis complications, electrolyte disturbances, end stage renal disease (esrd), general nephrology, severe hyperparathyroidism, intermittent hemodialysis

## Abstract

Calcific uremic arteriolopathy (CUA) or calciphylaxis is a rare disorder involving painful necrosis of the skin secondary to calcification and thrombosis of small arterioles in patients with chronic kidney disease (CKD) or end-stage renal disease (ESRD). Such events may even occur after parathyroidectomy (PTX), typically in the setting of adynamic bone disease and very low post-PTX parathyroid hormone (PTH) levels. In this report, we describe a 40-year-old woman with ESRD from IgA nephropathy who developed diffuse, painful subcutaneous calcifications that progressed gradually over several weeks, about 10 months after PTX for severe secondary hyperparathyroidism, evolving into a spontaneous ulceration on the left lateral thigh. Pre-PTX, she had a PTH value of 1389.6 pg/mL, but serum corrected calcium was only 7.3 mg/dL. Her postoperative course was complicated by worsening hypocalcemia, a prolonged need for intravenous calcium infusion after PTX consistent with hungry bone syndrome, and ongoing hyperphosphatemia with phosphorus persistently ranging from 6 to 8 mg/dL. Hungry bone-associated hypocalcemia typically requires weeks to months to stabilize, as was the case with this patient, and supplementation is generally titrated to maintain calcium in the low-normal range while avoiding excessive calcium loading. This patient continues to have chronic pain and poor wound healing despite numerous therapies. This case is intended to show the paradox of calciphylaxis in low PTH states and demonstrate the complex interplay of suppressed bone turnover and calcium dysregulation.

## Introduction

Calciphylaxis is a rare but potentially fatal complication that occurs in a minority of end-stage renal disease (ESRD) patients undergoing dialysis [1-3]. It presents with painful nodular skin ulcerations and necrosis, and the mortality has been reported to be in excess of 50% [2]. Incidence is estimated at 1-4% among dialysis patients, with ulcerated lesions carrying the highest mortality [1-3]. Hyperparathyroidism and hypercalcemia have been classically implicated in its pathogenesis. There is growing recognition, however, of cases developing after parathyroidectomy (PTX) or in the context of adynamic bone disease, where the post-PTX parathyroid hormone (PTH) levels are markedly decreased [4]. Under these conditions, reduced bone turnover limits skeletal buffering capacity for calcium and phosphate, favoring soft-tissue and vascular deposition. Other risk factors like female sex, obesity, diabetes, glucocorticoid use, excess dosages of activated vitamin D (or vitamin D analogs), and calcium-containing phosphate binders can also increase susceptibility to vascular calcification in this patient population [5,6].

## Case presentation

A 40-year-old White woman with ESRD due to biopsy-proven IgA nephropathy, on maintenance hemodialysis since 2021, was referred for evaluation of a painful left lateral thigh ulcer with suspicion of calciphylaxis. She had secondary hyperparathyroidism, chronic hypocalcemia, ESRD-associated anemia, hypertension, hyperlipidemia, gout, hypothyroidism, obesity with a peak weight of 396 lbs, and vitamin D deficiency. She had a subtotal PTX in August 2024 for severe secondary hyperparathyroidism. Operative documentation listed a “subtotal PTX,” but specific details regarding gland counts or auto-transplantation were not available in the chart. Preoperative PTH was 1389 pg/mL; however, her simultaneous calcium level was relatively low at 7.3 mg/dL. Her postoperative course was complicated by profound hypocalcemia with a calcium nadir of 5.9 mg/dL, ultimately requiring a month-long hospital stay for continued intravenous calcium infusions in a pattern consistent with hungry bone syndrome. Earlier outpatient PTH levels prior to the preoperative value were not available, limiting assessment of the chronicity of hyperparathyroidism.

Postoperatively, her PTH remained suppressed at <20 pg/mL through October 2025; a detailed review of key laboratory parameters post-PTX and those during clinic presentation is shown in Table 1. She received hemodialysis with a high-calcium bath post-PTX. Her calcium bath was later reduced after calciphylaxis was suspected. A normal calcium level was difficult to achieve, and phosphate control remained suboptimal at 6-8 mg/dL. In May of 2025, about nine months postoperatively, she began developing painful, progressive nodules across her thighs and calves that later ulcerated on the left thigh. Pain was severe, refractory to usual wound care, and greatly impacted her quality of life.

**Table 1:** Key laboratory trends before and after PTX. PTX: parathyroidectomy; PTH: parathyroid hormone; op: operative; ref: reference range; N/A: not available; L: low; LL: very low; H: high relative to reference range

Date	Clinical Phase	Calcium (mg/dL) (Ref: 8.5-10.2)	Phosphorus (mg/dL) (Ref: 2.5-4.5)	PTH (pg/mL) (Ref: 15-65)	Alkaline Phosphatase (U/L) (Ref: 44-147)	Magnesium (mg/dL) (Ref: 1.7-2.4)
7/24/2024	Pre-PTX baseline	7.4 (L)	N/A	N/A	205 (H)	N/A
8/6/2024; 7:23	Pre-op (day of PTX)	7.3 (L)	N/A	1389.6 (H)	158 (H)	N/A
8/6/2024; 11:01	Peri-op	6.9 (L)	N/A	120.8 (H)	N/A	N/A
8/7/2024; 5:01	Post-op nadir	5.9 (LL)	N/A	13	259 (H)	N/A
8/28/2024	Late post-op	6.5 (LL)	N/A	15.8	196 (H)	N/A
8/1/2025	12 months post-PTX	N/A	N/A	N/A	59	N/A
10/2/2025	Presentation with calciphylaxis	8.9	7.0 (H)	17.3	N/A	2.3

**Figure 1:**
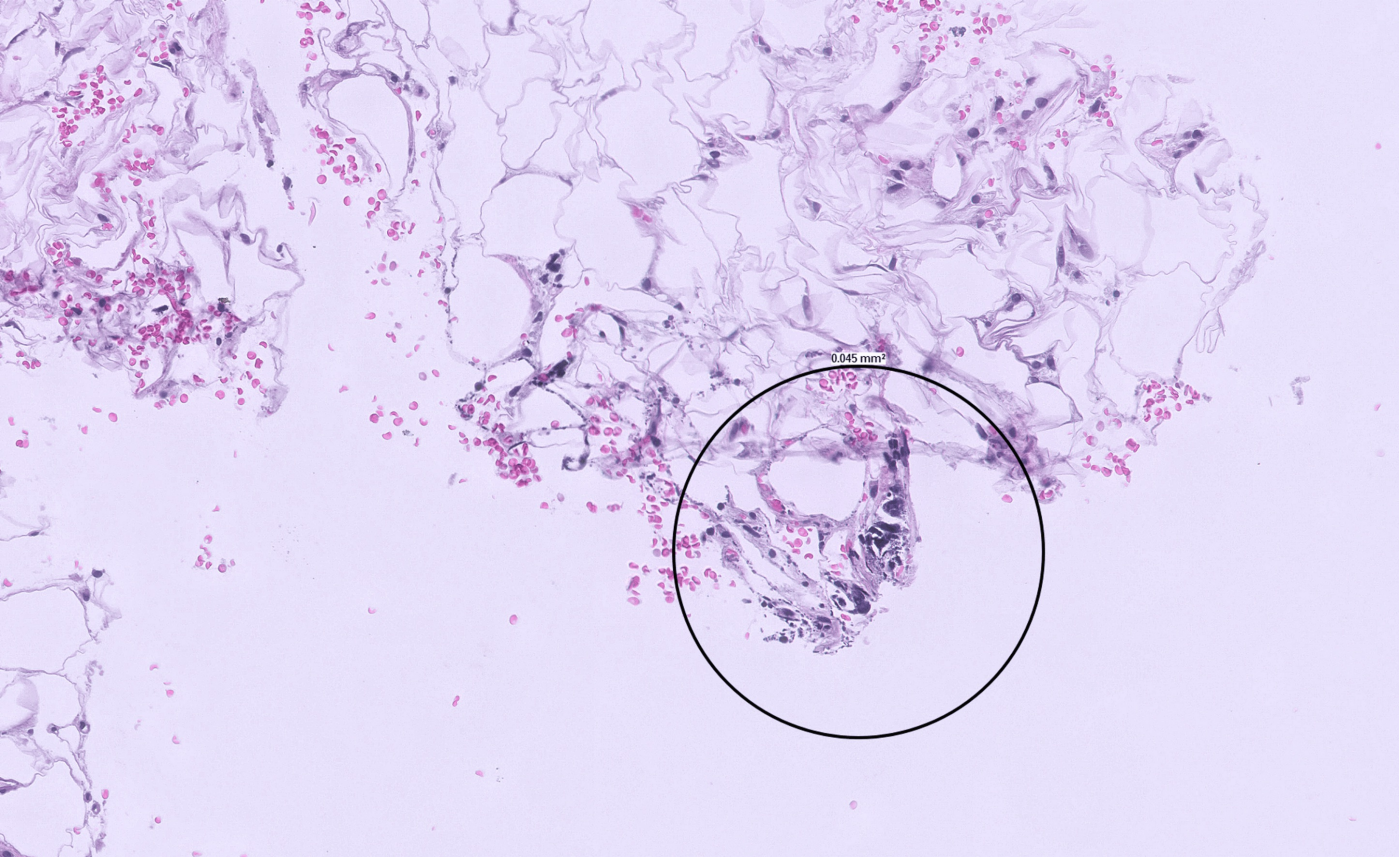
Skin biopsy from the left lateral thigh demonstrating focal subcutaneous calcification (hematoxylin and eosin stain, 200×). The deep subcutaneous tissue contains coarse, basophilic deposits consistent with calcified fat necrosis (circled). The surrounding adipose tissue is largely unremarkable, with minimal inflammatory infiltrate.

**Figure 2:**
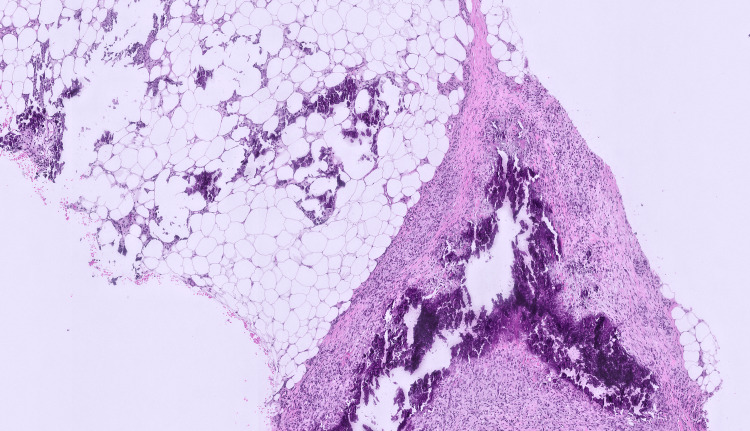
Right calf skin biopsy demonstrating calcified fat necrosis within the subcutaneous tissue (hematoxylin and eosin stain, 50×).

**Figure 3:**
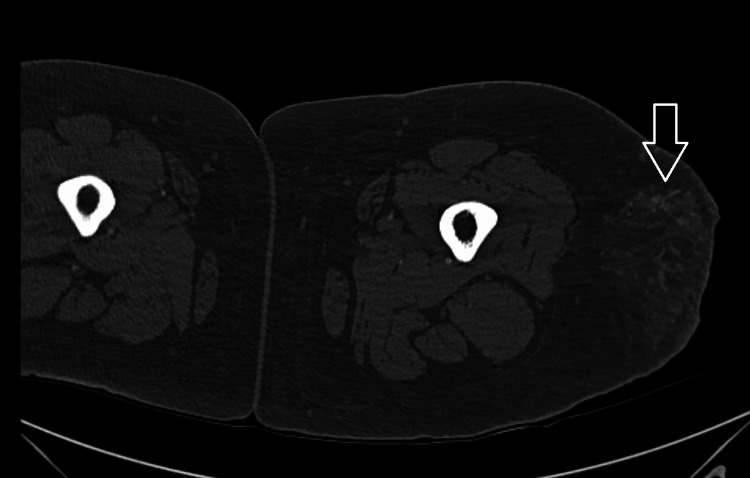
Computed tomography (CT) of the left femur without contrast demonstrating subcutaneous edema with scattered calcifications. The arrow indicates the area of calcification.

The first biopsy of one of the skin lesions in June 2025 revealed only mild perivascular inflammation without any feature of lupus panniculitis, lymphocytic lobular panniculitis, hyaline fat necrosis, or malignancy. The second biopsy (Figure 1) in August 2025 demonstrated calcified fat necrosis in subcutaneous tissue, which was diagnostic of calciphylaxis. She also underwent biopsy of the right calf (Figure 2). Subsequent computed tomography (CT) of the left thigh in October 2025 (Figure 3) showed ill-defined subcutaneous calcifications without any abscess or gas, which was consistent with calcific uremic arteriolopathy (CUA). Based on her skin lesions, chronic pain, imaging findings, and biopsy results, she presented to us for a second opinion and medical management of CUA.

Her medications upon presentation included calcitriol 0.5 µg twice daily, calcium citrate 800 mg/day, sucroferric oxyhydroxide, prednisone 20 mg/day initiated in May 2025 for pain, ergocalciferol 50,000 IU/week, levothyroxine, losartan, amlodipine, buspirone, sucralfate, and oxycodone. She was known to be sensitive to doxercalciferol and calcitriol but could tolerate low doses. She denied warfarin, vitamin K antagonist, or nonsteroidal anti-inflammatory drug (NSAID) use. Documentation did not specify whether calcium carbonate had been trialed prior to hospitalization, nor whether higher-dose oral calcitriol attempts were ineffective or poorly tolerated.

On examination, her blood pressure was 148/99 mmHg, and her body mass index (BMI) was 38.8 kg/m^2^. She appeared uncomfortable due to significant thigh pain but remained alert, oriented, and hemodynamically stable. There was a 3-cm ulcer with a violaceous, indurated center and borders and a yellow-gray base over the left lateral thigh with tender nodules surrounding it; similar nodules were present on both calves. No edema or additional dermatologic lesions, including macules, papules, or plaques, were noted. Her cardiovascular and pulmonary exams were unremarkable.

After our initial assessment in the clinic, the preponderance of clinical evidence suggested clinical CUA, and a multi-prong approach was warranted to avert further deterioration and assist healing from the current wound. Our recommendations included weaning of glucocorticoid therapy as soon as possible, limiting the dose of calcitriol to the minimum necessary to maintain low-end normal calcium level, with supplementation guided primarily by biochemical levels rather than clinical symptoms, aggressive medical control of hyperphosphatemia, and oral supplementation of magnesium and K_2_ vitamin.

Her early management included the minimization of calcium and vitamin D supplements and the reduction of pro-calcific stimuli. Prednisone taper was strongly recommended by 5 mg weekly with a plan to discontinue over one month. Calcitriol was reduced to 0.5 µg three times a week, and calcium gluconate was to be taken with meals for phosphate binding, with the goal of maintaining calcium 8.2-8.5 mg/dL. Non-calcium-containing phosphate binders were recommended, and this information was conveyed to the patient’s regular ESRD provider. Magnesium oxide was started to titrate serum magnesium to around 2.5 mg/dL. A summary of her medication changes and rationales for these changes is shown in Table 2.

**Table 2:** Medication changes and rationale for therapy modification. IV: intravenous; BID: twice daily; TID: three times daily; PTH: parathyroid hormone; ESRD: end-stage renal disease; NSAIDs: nonsteroidal anti-inflammatory drugs

Medication at Presentation	Revised Plan	Rationale
Prednisone 20 mg daily	Taper by 5 mg weekly, plan to discontinue within one month.	Glucocorticoids are implicated in protein catabolism, endothelial injury, and vascular calcification. De-escalation helps to lower pro-calcific activity.
Calcitriol 0.5 µg BID	Reduce to 0.5 µg three times weekly.	Excess active vitamin D suppresses PTH and promotes adynamic bone disease. Lowest dose to maintain Ca 8.2-8.5 mg/dL
Calcium citrate 800 mg/day	Replace with calcium gluconate. Taken with meals as a phosphate binder.	Shifts calcium use from supplementation to phosphate binding
Sucroferric oxyhydroxide (Ca-free binder)	Continue; consider adding sevelamer carbonate or lanthanum carbonate for improved phosphate control.	A combination of non-calcium binders helps reduce hyperphosphatemia without increasing calcium load.
Magnesium	Start magnesium oxide 400 mg BID/TID, goal Mg 2.5 mg/dL.	Magnesium inhibits vascular calcification and stabilizes calcium homeostasis.
Vitamin K_2_	Start menaquinone-7 1-2 mg/day, adjusted to 5 mg/week based on availability.	Vitamin K_2_ activates matrix Gla protein. Deficiency in this fat-soluble vitamin is common after bariatric surgery.
Sodium thiosulfate	Initiate IV sodium thiosulfate 12.5 g after dialysis 2-3×/week (or 25 g once-twice weekly if limited).	Chelates calcium, standard adjunct therapy for calciphylaxis. Effectiveness is largely in those with elevated ionized calcium - need to avoid excessive doses in the absence of hypercalcemia.
Oxycodone	Continue; refer to pain management; avoid NSAIDs.	Adequate analgesia required.

For the treatment of possible vitamin K deficiency, oral menaquinone-7 (vitamin K_2_) at 1-3 mg/day was recommended and, based on preparation availability in our area, this was revised to 50 mg/week for the activation of matrix Gla protein, an inhibitor of vascular calcification [1,2,4]. Sodium thiosulfate infusions, 12.5 g two to three times/week, or 25 g once/twice/week if logistically constrained, were started after dialysis sessions for calcium chelation. Pain was managed with opioids while awaiting referral to a pain specialist. She was also referred to a tertiary wound-care program. She remains under multidisciplinary care with nephrology, wound care, nutrition, and pain management.

## Discussion

Calciphylaxis involves calcification and thrombosis of skin arterioles, resulting in ischemic necrosis of skin and subcutaneous tissue. The pathogenesis is complex and multifactorial, centering on deranged mineral metabolism and loss of natural inhibitors of calcification [1,2]. Although classically linked with states of elevated PTH, many reports now document calciphylaxis after PTX [7-10]. Over-suppression of PTH and the evolution of adynamic bone disease can reduce skeletal buffering of calcium and phosphate, with soft tissue deposition of these ions [5,6]. The acute reduction in our patient’s PTH from more than 1300 pg/mL to less than 20 pg/mL, effectively a non-detectable level consistent with near-complete parathyroid ablation, occurred in the setting of vigorous intravenous calcium replacement and persistent hyperphosphatemia with phosphorus levels of 6 to 8 mg/dL, creating calcium-phosphate supersaturation that most likely precipitated her CUA. In addition to proper patient selection, intraoperative PTH monitoring is important to avoid excessive suppression of parathyroid tissue, although the operative details in this case were limited.

In normal physiology, PTH secretion is under tight control by the calcium-sensing receptor in a feedback system whereby hypocalcemia causes and hypercalcemia suppresses secretion of PTH. Thus, even a seemingly high level, like in our index case, may not represent elevated bone reformation when PTH secretion is stimulated by hypocalcemia and the normal reference range is modified by the presence of CKD. Moreover, in CKD, the calcium-PTH set point is altered, and after PTX, the adaptive response is eliminated, resulting in a tendency to long-term hypocalcemia and adynamic bone disease [9]. Ionized calcium levels were not available in the record, which limits assessment of physiologic calcium activity.

Adynamic bone disease is a low bone turnover state with depressed osteoblastic activity, typically due to excess vitamin D analogs, calcium binders, or surgical hypoparathyroidism [5,6]. The skeleton fails to take up excess calcium and phosphate, raising their serum concentration and promoting vascular calcification. Laboratory findings are low PTH, low alkaline phosphatase, low-normal calcium, and sustained hyperphosphatemia [4-6].

Diagnosis of CUA is made through a combination of clinical suspicion, biopsy confirmation, and imaging [11,12]. Laboratory abnormalities alone are nonspecific. Histology showing calcified fat necrosis is diagnostic, whereas radiologic findings of subcutaneous calcifications are supportive [12,13]. Bone scintigraphy may be useful when biopsy is nondiagnostic, but it was not performed in this case because histologic confirmation was already obtained [11-13]. Calciphylaxis can occur in the setting of suppressed PTH and low or normal calcium levels after PTX [3-6]. The low PTH variant is associated with adynamic bone disease and often worsens with treatment with calcium and vitamin D [5,6]. This patient had a sleeve gastrectomy in 2021, which was likely a contributing factor for malabsorption of fat-soluble vitamins. Reduced hydrochloric acid secretion after gastrectomy may have also impaired intestinal calcium absorption and reduced the efficacy of calcium as an intestinal phosphorus binder [14]. Deficiency of vitamin K, particularly after bariatric surgery, a known risk factor for fat malabsorption and reduced availability of fat-soluble vitamins, may play an underappreciated role and warrants further investigation. Recognition and elimination of inciting causes early can improve prognosis, although once established, the disease process is difficult to reverse [2,8,15].

## Conclusions

Calciphylaxis is one of the most serious complications of mineral imbalance in ESRD. This case illustrates that post-PTX oversuppression of PTH, in combination with heavy calcium-phosphate load, and possibly vitamin K deficiency, can contribute to CUA even in the context of surgical hypoparathyroidism. Clinicians should be vigilant for painful skin lesions in dialysis patients post-PTX and reassess calcium, phosphate, and vitamin D therapy in a timely fashion to prevent this painful and potentially lethal complication.
